# Selection of *Lactiplantibacillus* Strains for the Production of Fermented Table Olives

**DOI:** 10.3390/microorganisms10030625

**Published:** 2022-03-15

**Authors:** Teresa Zotta, Marilisa Giavalisco, Eugenio Parente, Gianluca Picariello, Francesco Siano, Annamaria Ricciardi

**Affiliations:** 1 Scuola di Scienze Agrarie, Alimentari, Forestali ed Ambientali (SAFE), Università degli Studi della Basilicata, 85100 Potenza, Italy; teresa.zotta@unibas.it (T.Z.); marilisa.giavalisco@unibas.it (M.G.); annamaria.ricciardi@unibas.it (A.R.); 2 Istituto di Scienze dell’Alimentazione-CNR, 83100 Avellino, Italy; gianluca.picariello@isa.cnr.it (G.P.); francesco.siano@isa.cnr.it (F.S.)

**Keywords:** *Lactiplantibacillus* *plantarum*, *Lactiplantibacillus* *pentosus*, phenolic compound tolerance, technological characterization, gene occurrence

## Abstract

*Lactiplantibacillus* strains (n. 77) were screened for technological properties (e.g., xylose fermentation, EPS production, antimicrobial activity, tolerance to NaCl and phenolic compounds, oleuropein degradation and hydroxytyrosol formation) relevant for the production of fermented table olives. Survival to olive mill wastewater (OMW) and to simulated gastro-intestinal tract (GIT), the capability to grow at different combinations of NaCl and pH values, radical scavenging activities and biofilm formation were further investigated in 15 selected strains. The screening step revealed high diversity among *Lactiplantibacillus* strains. Most of the strains were able to ferment xylose, while only a few strains produced EPS and had inhibitory activity against *Y. lipolytica.* Resistance to phenolic compounds (gallic, protocatechuic, hydroxybenzoic and syringic acids), as well as the ability to release hydroxytyrosol from oleuropein, was strain-specific. OMWs impaired the survival of selected strains, while combinations of NaCl ≤ 6% and pH ≥ 4.0 were well tolerated. DPPH and hydroxyl radical degradation were strain-dependent, while the capability to form biofilm was affected by incubation time. Strains were very tolerant to the GIT. The genome of *Lpb. pentosus* O17 was sequenced and analysed to verify the presence of genes involved in the degradation and metabolism of phenolic compounds. O17 lacks carboxylesterase and gallate decarboxylase (subunits B and D) sequences, and its gene profile differs from that of other publicly available *Lpb. pentosus* genomes.

## 1. Introduction

*Lactiplantibacillus paraplantarum*, *Lpb. plantarum* and *Lpb*. *pentosus* (formerly *Lactobacillus paraplantarum*, *Lb. plantarum* and *Lb. pentosus* [1]) are three phylogenetically close species [1] involved in several food, biotechnological and health-related applications. Strains belonging to the *Lactiplantibacillus* group have a wide ecological distribution (e.g., presence in vegetable, cereal, meat and dairy products, as well as human hosts) and are characterized by high phenotypic and genomic diversity. *Lactiplantibacillus* genomes, in fact, are large (from 2.9 to 5.3 Mbp; NCBI database), and comparative genome and pangenome analyses revealed the presence of accessory and unique (strain-specific) genes consistent with the evolution and adaptation to different ecological niches, confirming the versatility of this group [2,3,4].

Strains of the *Lactiplantibacillus* group are important members of olive surface microbiota [5,6] and, thus, they can play a crucial role in the production of olive-derived products. Fermented table olives can be processed with different methods: drupes can be debittered through an alkaline treatment (NaOH 2.5–3% *w*/*v*) and then fermented in brine (NaCl 10–11% *w*/*v*) for 3–7 months (treated table olives, Spanish style); alternatively, olives can be fermented directly in brine (NaCl 6–10% *w*/*v*) for 8–12 months, with a debittering process driven by the enzymatic activities of indigenous microorganisms (natural table olives, Greek style; see Perpetuini et al. [5] for more details).

*Lactiplantibacillus* strains may dominate the spontaneous fermentation [7,8,9] of several types of table olives, or they may be deliberately added as starter and adjuncts, alone or in combination with yeasts, to control and standardize the fermentative process [5,10,11]. *Lactiplantibacillus* starters may reduce the fermentation time, and may improve the organoleptic (e.g., debittering, aroma profile, pulp and colour stability), nutritional (e.g., antioxidant compounds) and microbiological quality (e.g., the acidification of brine and antimicrobial compounds may prevent spoilage and pathogens) of table olives [5].

The survival of strains (naturally present or deliberately added) during the fermentative process (both in Spanish- and Greek-style olives) depends on several factors (e.g., low pH, high concentrations of salt and phenolic compounds and competition with other species) and, therefore, the capability to cope with multiple stresses is essential for their fitness during fermentation and for obtaining stable and high-quality products.

The presence of high phenolic compound levels is certainly one of the main constraints in olive fermentation. Members of the *Lactiplantibacillus* group are able to degrade and use several phenolic compounds (e.g., gallic, *p-*coumaric, ferulic, protocatechuic acids and oleuropein), although this feature is strain-dependent and has been often investigated in in vitro studies (i.e., in synthetic media containing phenolic compounds) [12]. Moreover, many studies have addressed the response to single phenolic compounds, but it is known that olives are a complex phenol-rich matrix in which the synergic effect of different phenolic fractions may exacerbate their antimicrobial activity. In brines, additionally, the resistance to phenolic compounds (released in aqueous phase) is also affected by the presence of high salt concentrations and low pH values [13]. The metabolism of phenolic compounds in the *Lactiplantibacillus* group is related to the activity of several degrading enzymes (e.g., β-glucosidase, esterase, tannase, decarboxylases), and the occurrence of genes coding for these enzymes may be a criterion for the selection of strains. To date, the occurrence of genes has been mainly evaluated in *Lpb. plantarum* strains and, to a lesser extent, in *Lpb. pentosus* [14,15,16,17,18], although this species is frequently isolated and associated with olives and their derived products [5,13].

In this study, we tested a collection of *Lactiplantibacillus* strains (n. 77, isolated from different sources) for technological properties (i.e., xylose fermentation, EPS, GABA and catalase production, antimicrobial activity, tolerance to NaCl and phenolic compounds, oleuropein degradation and hydroxytyrosol formation) useful for the production of fermented olives. Particular efforts focused on the tolerance against phenolic compounds, including the survival in olive mill wastewater (selected as a model of a phenol-rich matrix). The genome of *Lpb. pentosus* O17 (the most tolerant and promising strain) was sequenced and analysed to verify the occurrence of genes involved in the degradation and metabolism of phenolic compounds, and the gene profile was compared with that of other publicly available *Lpb. pentosus* genomes.

## 2. Materials and Methods

### 2.1. Strains and Culture Conditions

Seventy-seven strains of *Lactiplantibacillus paraplantarum* (n. 4), *Lpb. plantarum* (n. 50) and *Lpb*. *pentosus* (n. 23) were used in this study (Table 1). The strains were isolated from different sources and maintained as freeze-dried stocks (11% *w*/*v* skim milk with 0.1% *w*/*v* ascorbic acid) in the Culture Collection of Industrial Microbiology Laboratory, Università degli Studi della Basilicata. Lactobacilli were propagated (16 h, 30 °C) in Weissella Medium Broth pH 6.8 (WMB [19]) before each assay.

The strains *Debaryomyces hansenii* DH-02, *Yarrowia lipolytica* YL-12*, Listeria innocua* BL86/26 and *Lpb. plantarum* C17 were used as indicators for the deferred antagonism assay (Section 2.3). *D. hansenii* and *Y. lipolytica* were routinely cultivated (24 h, 25 °C) in yeast extract–Peptone–dextrose broth (YPD broth), *L. innocua* in tryptone soya broth supplemented with 6 g/L yeast extract (TSBYE; 24 h, 30 °C) and *Lpb. plantarum* C17 in WMB (16 h, 30 °C).

### 2.2. Identification of Lactiplantibacillus Strains Isolated from Olives and Brines

Sixty-five strains were already present in the Culture Collection of Industrial Microbiology, while twelve *Lactiplantibacillus* (* in Table 1) were isolated, in this study, from natural and cured (NaOH treatment) table olives and the brines of *Bella di Cerignola* and *Cellina di Nardò* (Puglia, Italy) cultivars. Specifically, 24 isolates were recovered (30 °C, 24 h, anaerobiosis) on modified MRS agar (mMRS) supplemented with 0.02 g/L bromophenol blue and 0.5 g/L cysteine (for colony morphology [20]) and with 100 mg/L cycloheximide (to inhibit growth of yeasts and moulds). Specifically, light-blue colonies with dark-blue centres (morphology of *Lactiplantibacillus* strains on mMRS [20]) were collected and purified before taxonomic identification.

Genomic DNA was extracted from each isolate by using the GeneElute Bacterial Genomic DNA Kit (Sigma-Aldrich, St. Louis, MO, USA) and quantified with a NanoDrop 1000c spectrophotometer (Thermo Scientific, Wilmington, DE). Isolates were de-duplicated using RAPD-PCR (primer M13 [21]) and identified at species levels through amplification (Table S1) and sequencing (external service, Genechron Srl, Roma, Italy) of the *16S rRNA* gene and with a multiplex PCR assay (amplification of *recA* gene [22]).

### 2.3. Technological Characterization of Lactiplantibacillus Strains: A Screening Step

All strains were tested for technological features that could be useful for the production of fermented table olives and for other applications related to olive-derived products.

Xylose fermentation: WMB cultures were plated on MRS agar containing 10 g/L D-xylose and 0.16 g/L bromocresol purple (BCP) as the pH indicator, and incubated at 30 °C for 48 h under anaerobic conditions. The change of substrate colour from purple to yellow and the presence of yellow colonies indicated the ability to use xylose.

Tolerance of high NaCl concentrations: WMB cultures were standardized to a final absorbance at 650 nm (A_650_) of 1.0 (Bio-Rad Smart Spec™ Plus, Bio-Rad Laboratories Inc.) and used to inoculate (10% *v*/*v*) WMB supplemented with 7.5% *w*/*v* or 10% *w*/*v* NaCl and 0.16 g/L BCP. After incubation (30 °C, 48 h, anaerobiosis) the change of colour from purple to yellow indicated the presence of survivors.

Production of exopolysaccharides (EPS): the strains were screened for EPS production by using a pick test on MRS agar plates containing 20 g/L maltose (M-MRS) or 20 g/L glucose (G-MRS) or 50 g/L sucrose (S-MRS) as carbon sources. At the end of incubation (30 °C, 24 h, anaerobiosis), the strains which produced translucent slimy or ropy colonies were recorded as EPS producers.

Production of γ-amino butyric acid (GABA): WMB cultures were standardized (absorbance at 650 nm, A_650_ of 3.0) and used to inoculate (1% *v*/*v*) WMB with or without 1% (*w*/*v*) monosodium glutamate (MSG). At the end of incubation (30 °C, 24 h, anaerobiosis), pH values (CyberScan pH11/110, Oakton Instruments, Vernon Hills, USA; Double Pore Slim electrode, Hamilton Company, Reno, NV, USA) and A_650_ were measured, and the supernatants were collected (12,000× *g*, 5 min, 4 °C). The accumulation of GABA in unsupplemented WMB and WMB-MSG was qualitatively evaluated by thin layer chromatography (TLC; silica gel plates, cod. Z292974-1PAK, Sigma-Aldrich, St. Louis, MO, USA). The mobile phase was a mixture of *n*-butanol, acetic acid and deionized water (4:1:1 ratio), supplemented with 0.2% (*w*/*v*) ninhydrin for spot detection (red-purple colour). Samples were loaded (1.5 µL/spot) on TLC plates and separated in a TLC chamber at room T°C; after the run, the TLC plates were heated at 70 °C for spot visualization. WMB supplemented with 1% (*w*/*v*) MSG or GABA were used as controls.

Antimicrobial activity: inhibitory activity was tested by using a deferred antagonism assay, as described in Parente et al. [23]. All lactobacilli were spotted (5 μL) on MRS agar plates and incubated at 30 °C for 48 h. The overlay soft-agar (0.6% *w*/*v*) media containing the indicator strains were YPD for *D. hansenii* DH-02 and *Y. lipolytica* YL-12, TSBYE for *L. innocua* BL86/26 and buffered MRS (0.1 mol/L MOPS) for *Lpb. plantarum* C17. At the end of incubation (24 h, 30 °C), the diameter of the inhibition zones around spots were measured using a calliper.

Catalase activity: the catalase activity of lactobacilli cultivated in unsupplemented and heme-supplemented WMB was qualitatively detected (bubble formation) as reported by Zotta et al. [24].

Resistance to phenolic compounds: the strains were cultivated (30 °C, 24 h) in modified WMB pH 6.5 containing 2 g/L of ribose as a carbon source (mWMB), washed twice with NaCl 0.85% (*w*/*v*), standardized to an A_650_ of 1.0 and used to inoculate (10% *v*/*v*; 96-well-microplate experiments) mWMB supplemented with gallic acid (two-fold dilution from 100 to 6.25 mM), protocatechuic acid (from 50 to 3.12 mM), 4-hydroxybenzoic acid (from 40 to 2.5 mM) and syringic acid (from 25 to 1.56 mM). After incubation (30 °C, 24 h), the substrate turbidity (semi-quantitative assay) indicated the presence of survivors at a given concentration of phenolic compound.

Hydrolysis of oleuropein and production of hydroxytyrosol: standardized mWMB cultures (as above) were used to inoculate (10% *v*/*v*) 96-well microplates containing MRS pH 7.4 (without meat extract [25]) supplemented with 1 g/L oleuropein. At the end of incubation (7 days, 30 °C, anaerobiosis), the supernatants were collected (12,000× *g*, 5 min, 4 °C) and the oleuropein degradation and hydroxytyrosol formation were qualitatively evaluated through a TLC assay, as described by Ciafardini et al. [25], with some modifications. Briefly, the mobile phase was a mixture of *n*-propanol: benzyl alcohol: 88%; formic acid: water (50:72:20:20 ratio). Samples were loaded (1.5 µL/spot) on TLC plates, and separated in a TLC chamber at room T°C. After the run, the TLC plates were dipped in Folin–Ciocâlteu 30% (*v*/*v*) and air-dried at room T°C for spot visualization (grey-brown colour). MRS pH 7.4 supplemented with oleuropein or hydroxytyrosol (0.25 g/L final concentration) were used as controls. For each assay, two biological replicates were carried out.

### 2.4. Characterization of Selected Lactiplantibacillus Strains

Fifteen strains (*Lpb. pentosus* OM13, OM14, OM24, OM50, OM53, OM52, OM62, 2TP, O17 and O18; *Lpb. plantarum* subsp. *plantarum* C17, ISLCPT57, MT2A11S and WCFS1; *Lpb. plantarum* subsp. *argentoratensis* MTC13L) were selected on the basis of previous assays, routinely cultivated in WMB (30 °C, 24 h) and used to verify for the following properties:

Radical scavenging activity: the capability to remove 1,1-diphenyl-2-picrylhydrazil (DPPH) and hydroxyl radicals was measured on standardized cells (A_650_ = 1.0), as described by Wang et al. [26]. For both assays, two biological replicates were carried out.

Biofilm formation: standardized cells (A_650_ = 1.0) were used to inoculate 96-well (flat-bottomed) polystyrene microplates, filled with WMB pH 6.8. After 24 and 48 h of incubations at 30 °C, supernatants and non-adherent cells were removed by inverting the plates, and biofilms were stained with 200 μL of crystal violet (0.1% *w*/*v*) for 30 min at 30 °C ([27], with some modifications). At the end of the incubation, the excess dye was discarded and gently washed (3 times) with phosphate buffer 20 mM pH 7.0 (PB7). Crystal violet was solubilized in 200 μL of 96% (*v*/*v*) ethanol, and absorbance at 595 nm (A_595_) was measured with a microplate reader (SPECTROStar Nano; BMG Labtech, Ortenberg, Germany). Three biological replicates were carried out.

Resistance to different combinations of NaCl and pH values: standardized cells (A_650_ = 1.0) were used to inoculate (10% *v*/*v*; 96-well microplates) WMB with different combinations of NaCl (0, 4.0, 6.0 and 8.0% *w*/*v*) and pH (6.0, 5.0, 4.0 and 3.0) values. WMB pH 6.0, without salt, was used as the control. After 24 and 48 h of incubations at 30 °C, A_650_ values were measured with a microplate reader. Two biological replicates were carried out.

Resistance to simulated gastro-intestinal tract: standardized cells (A_650_ = 1.0) were re-suspended in simulated saliva (SS; 25 mM NaCl, 7 mM KCl, 45 mM NaHCO_3_, 100 mg/L lysozyme pH 6.9 [28]) and incubated at 100 rpm (SW20 shaking thermostatic water bath, JULABO GmBH, Seelbach, Germany) for 5 min at 37 °C. At the end of the incubation, cells were harvested (8000× *g*, 10 min, 4 °C), re-suspended in simulated gastric juice (SGJ; 25 mM NaCl, 7 mM KCl, 45 mM NaHCO_3_, 0.3 g/100 mL pepsin, pH 3.0 [29]) and incubated at 100 rpm for 1 h at 37 °C. SGJ-treated cells were then collected, re-suspended in simulated pancreatic juice (SPJ; 0.15 g/100 mL bile salt, 0.1 g/100 mL trypsin, 0.1 g/100 mL α-chymotrypsin, pH 8.0 [29]) and incubated at 100 rpm for 1 h at 37 °C. Untreated cells, incubated in PB7, were used as controls. The numbers of survivors were enumerated in each step (SS, SGJ and SPJ) by pour plating on WMB agar (30 °C, 48 h, anaerobiosis). Two biological replicates were carried out.

Survival in olive mill wastewater: Olive mill wastewaters (OMWs) obtained from the olive oil production of *Leccino*, *Coratina* (oil mill located in Tricarico, Basilicata region, Italy) and *Cima di Melfi* (olive oil mill located in Grottaglie, Puglia region, Italy) cultivars were treated at 90 °C for 15 min and centrifuged (2800× *g*, 15 min, 20 °C) to remove solid residues. The effectiveness of heat treatment was verified via plate counting on different substrates (mMRS agar with 100 mg/L cycloheximide and 15 mg/L nalidixic acid, 48 h at 30 °C for detection of lactic acid bacteria (LAB); glucose–yeast extract–agar, GYEA, with 100 mg/L chloramphenicol, 30 °C at 48 h for yeasts and 5 days for moulds; VRBGA, 24 h at 30 °C for *Enterobacteriaceae*; gelatine peptone agar, 72 h at 30 °C for total aerobic count).

The strains were standardized and inoculated (final population of 10^7^ ufc/mL) in different concentrations (two-fold dilution from 50% to 0% *v*/*v*; 96-well-microplate experiments) of OMWs for 15 min at 30 °C. At the end of the incubation, OMW-treated cells were inoculated (10% *v*/*v*) in WMB broth pH 6.8 to evaluate the presence of survivors (substrate turbidity; semi-quantitative assay) after 24 h of incubation at 30 °C.

The strains with the highest resistance (*Lpb. pentosus* OM24 and O17, *Lpb. plantarum* C17 and MT2A11S) were further investigated for their tolerance to OMW (from 50% to 0% *v*/*v*) by using longer incubation times (15, 30, 60 and 120 min, at 30 °C). Survivors were evaluated as described before. For each assay, two biological replicates were carried out.

### 2.5. Total Phenolic Content and Phenolic Profile of OMW

OMW supernatants (3000× *g*, 15 min, 4 °C) of *Leccino*, *Coratina* and *Cima di Melfi* cv. (Section 2.5) were filtered (Millex^®^ filter 0.22 µm, Merck-Millipore, Billerica, MA, USA) and used for the detection of total phenolic content (TPC; Folin–Ciocâlteu assay) and the analysis of the phenolic profile (PP; HPLC-DAD).

For TPC, 50 µL of 40-fold-diluted filtered OMWs was combined with 2.3 mL deionized water and 50 µL Folin–Ciocâlteu 50% (*v*/*v*), and, after 3 min, 100 µL of saturated sodium carbonate was added. Mixtures were incubated in the dark at room T°C for 90 min. At the end of the incubation, absorbance at 765 nm (A_765_) was measured. A calibration curve (R^2^ > 0.99) with different concentration of gallic acid (from 0.05 g/kg to 0.5 g/kg) was used to quantify the TPC. The results were expressed as grams of gallic acid equivalent (GAE) per kilogram of OMW (g GAE/kg of OMW). Three technical replicates were performed for each sample.

For HPLC analysis, the filtered OMWs were 40-fold diluted with 0.1% (*v*/*v*) trifluoroacetic acid (TFA), and the phenolic compounds were separated using a modular chromatograph HP1100 (Agilent Technologies, Paolo Alto, CA, USA) equipped with a reverse phase C18 column, 250 × 2.0 mm i.d., 4 mm particle diameter (Jupiter Phenomenex, Torrance, CA, USA). The column temperature was held at 40 °C during the HPLC analysis. Analyses were performed at a constant flow rate of 0.2 mL/min, applying the following gradient of solvent B (acetonitrile/0.1% *v*/*v* TFA): isocratic elution at 5% of B for 5 min, and linear gradient 5–60% of B for 5–65 min and 60–100% of B at 65–70 min. Solvent A was 0.1% TFA in HPLC-grade water. For each run, 100 μL of diluted samples was injected. Analyses were performed in triplicate and monitored at 520, 360, 320, 254 and 280 nm, also acquiring the UV-Vis spectrum every second in the 200–600 nm range with a diode array detector (DAD). The main peaks were assigned according to the order of elution [30], matching the UV-Vis spectra with those of pure phenolics, with matches confirmed with authentic standard compounds if available. The oleuropein derivative was confirmed through the HPLC monitoring of its disappearance and the release of hydroxytyrosol upon acidic hydrolysis (3N HCl, 40 °C, 3 h). Chromatograms were analysed by using the Chem Station vers A.07.01 HPLC software (Agilent Technologies, Santa Clara, CA, USA).

### 2.6. Genome Sequencing of Lactiplantibacillus pentosus O17 and Occurrence Analysis of Genes Involved in Degradation and Metabolism of Phenolic Compounds

The whole genome of *Lpb. pentosus* O17, the strain with the best tolerance, was sequenced (WGS) by using an Illumina NovaSeq 6000 platform (IGA Technology s.r.l., Udine, Italy). The reads were de novo assembled using SPAdes v.3.14, while the functional annotation was performed using the NCBI Prokaryotic Genome Automatic Annotation Pipeline (PGAP). The WGS project was deposited at GenBank under the accession number JAHLCJ000000000.1 (https://www.ncbi.nlm.nih.gov/nuccore/JAHLCJ000000000.1 accessed on 15 January 2022).

The WGS of *Lpb. pentosus* O17 was analysed to verify the occurrence (presence, absence) of genes involved in the degradation and metabolism of phenolic compounds: *β*-glucosidase (also annotated as 6-phospho-*β*-glucosidase), gallate decarboxylase (subunits B, C, D), *p*-coumaric acid decarboxylase (also annotated as phenolic acid decarboxylase), esterase (also annotated as acetyl esterase/lipase or alpha/beta hydrolase), carboxylesterase, tannase (subunits A and B) and the transcriptional regulator PadR. The genes of interest were identified on the basis of data available in literature (see Table S2).

Gene profile of *Lpb. pentosus* O17 was compared with that of 28 *Lpb*. *pentosus* genomes retrieved from Integrated Microbial Genome database (IMG; https://img.jgi.doe.gov/cgi-bin/mer/main.cgi; accessed on 15 January 2022).

### 2.7. Statistical Analysis

Statistical analyses and graphs were obtained using Systat 13.0 for Windows (Systat Software Inc., San Jose, CA, USA) and R 4.1.2 [31].

## 3. Results and Discussion

### 3.1. Identification and Technological Characterization of Lactiplantibacillus Strains

In this study, a collection of *Lactiplantibacillus* strains were tested for some technological properties useful for the production of fermented table olives. The strains came from different isolation sources (Table 1) in order to verify possible correlations between functionality and ecological niches of isolation. In this study, 24 isolates were recovered from olives and brines, and multiplex PCR and partial *16S rRNA* gene sequencing confirmed their membership to *Lpb. plantarum* (n. 3) and *Lpb. pentosus* (n. 9) species.

**Figure 1:**
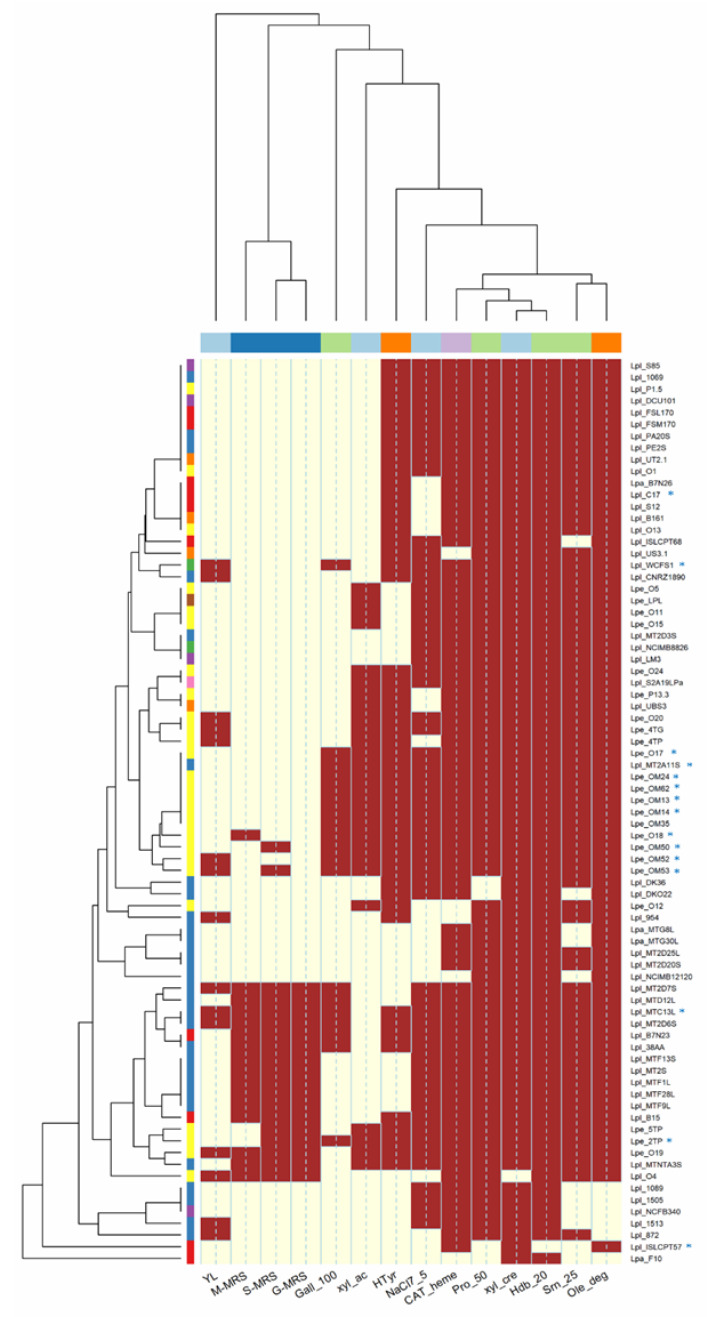
Correlation among technological properties of *Lactiplantibacillus* strains. Red box: positive; yellow box: negative. Code of columns of the dendrogram: YL, antimicrobial activity against *Yarrowia lipolytica*; M-MRS, S-MRS and G-MRS, production of exopolysaccharides on maltose, sucrose and glucose, respectively; Gall_100 (100 mM gallic acid), Pro_50 (50 mM protocatechuic acid), Hdb_20 (20 mM 4-hydroxybenzoic acid) and Srn_25 (25 mM syringic acid) referred to phenolic compound tolerance; xyl_cre, growth on xylose; xyl_ac, acid production from xylose; NaCl7_5, salt tolerance; CAT_heme, catalase activity; Ole_deg, oleuropein hydrolysis; Htyr, hydroxytyrosol formation. Rows of the dendrogram (similarity relationships among strains): LAB species: Lpl, *Lpb. plantarum*, Lpa, *Lpb. paraplantarum*, Lpe, *Lpb. pentosus*; isolation source: vegetables (violet), sourdoughs (blue), olives and derived products (yellow), dairy products (red), wine (orange), human sources (green), meat products (brown), unknown sources (pink).

All strains (with the exception of *Lpb. plantarum* O4) were able to grow in the presence of xylose (Figure 1), but only 27 were able to produce acid. This feature was widespread among isolates from olives (85%), although some strains from sourdoughs (i.e., MTNTA3S and MT2A11S) and wine (i.e., UBS3) also fermented xylose. This pentose is a hemicellulose-derived sugar present in several plant materials, including olive pulp, stones and pomace [32]. Therefore, the capability to use it may be of practical relevance in the production of olive-derived products. Additionally, D-xylose may be used to discriminate *Lactiplantibacillus* species, as the strains of *Lpb. paraplantarum* are unable to ferment it [33].

The ability to tolerate high salt concentrations is another relevant feature for the production of fermented olives. Several table olives (e.g., green Spanish style, black Greek style), in fact, are produced with brines containing from 4 to 12% of NaCl, which is useful to prevent spoilage and modify the organoleptic properties of drupes [5,11]. On the other hand, the salt concentration in brines significantly affects olive microbiota, increasing the relative abundance of yeasts compared to LAB at NaCl levels above 8% [6].

In this study, no strains were able to grow in the presence of 10% NaCl, while most of them (n. 58, mostly from olives and brines; Figure 1) grew with 7.5% NaCl. *Lpb. paraplantarum* strains, however, were very sensitive to salt, and were unable to grow at 7.5% NaCl. Several authors [25,34,35,36,37,38] have demonstrated that salt concentrations higher than 10% NaCl were detrimental for many *Lactiplantibacillus* strains, while lower levels (synthetic media supplemented with ≤ 7–8% NaCl) were well tolerated.

Sixteen strains (69% were from sourdoughs) produced EPS from both glucose, maltose and sucrose; *Lpb. pentosus* O18 formed EPS only from maltose, while *Lpb. pentosus* O19, OM50 and OM53 formed EPS only from sucrose (Figure 1; Table S3). No strain was able to produce dextran. The EPS from LAB may be used in the food industry as stabilizers, emulsifiers or gelling agents as they affect the rheological properties and texture of foods [39]. EPS, moreover, can be involved in adhesion and biofilm formation, improving the colonization ability of strains. EPS production in *Lactiplantibacillus* strains, then, may be beneficial to guarantee their permanence on olive fruits during fermentation.

The *Lactiplantibacillus* strains screened in this study did not show antagonistic activity against *Lpb. plantarum*, *D. hansenii* (members of olive microbiota [40]) and *L. innocua* (used as a proxy for *L. monocytogenes*, a pathogen possibly presents in table olives, especially under reduced salt fermentation [41,42]). Only 15 strains had inhibitory activity against *Y. lipolytica* (associated with olive microbiota; see Figure 1 and Table S3). The antimicrobial activity of *Lactiplantibacillus* isolated from olives (like other LAB) is related to the production of bacteriocins and organic acids [11,43,44], but these features are strongly affected by fermentation conditions (e.g., T°C, pH, salt concentrations [11]).

In this study, none of the strains were able to produce GABA when cultivated in the presence of sodium glutamate, while most of them (71, 92.2%) were able to synthesize heme catalase, the main H_2_O_2_-degrading enzyme in LAB.

### 3.2. Tolerance and Degradation of Phenolic Compounds

Olives and their derived products are phenolic-rich matrices, and the assessment of strain survival is crucial for the development of competitive cultures. Phenolic compounds detected in olives mainly include hydroxycinnamic (caffeic, *p*-coumaric, *o*-coumaric, ferulic and sinapic) and hydroxybenzoic (gallic, 4-hydroxybenzoic, protocatechuic, syringic and vanillic) acids, as well as phenolic alcohols, such as tyrosol and hydroxytyrosol [12,45].

**Figure 2:**
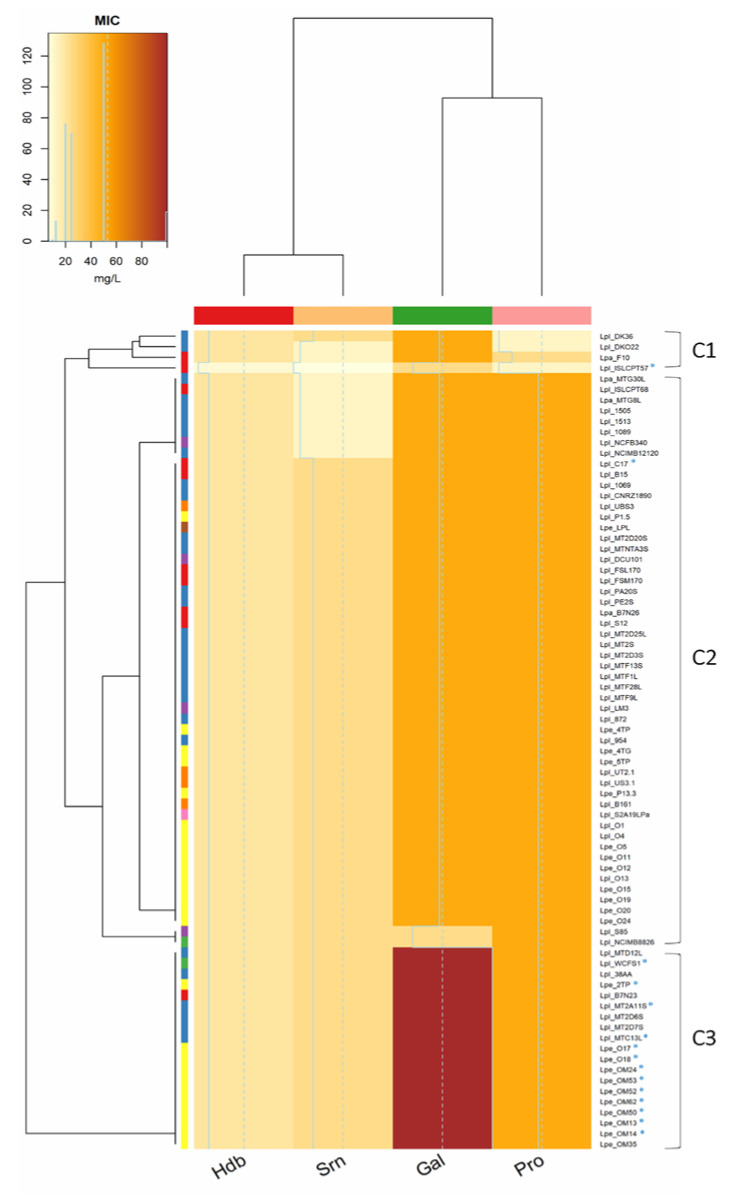
Minimum inhibitory concentration (MIC, mg/L) of 4-hydroxybenzoic (Hdb, 40–2.5 mM), syringic (Srn, 25–1.56 mM), gallic (Gal, 100–6.25 mM) and protocatechuic (Pro, 50–3.12 mM) acids. Phenolic compound concentration is defined by different colours shades, from yellow (0 mg/L) to red (100 mg/L). Rows of the dendrogram: LAB species: Lpl, *Lpb. plantarum*, Lpa, *Lpb. paraplantarum*, Lpe, *Lpb. pentosus*; isolation source: vegetables (violet), sourdoughs (blue), olives and derived products (yellow), dairy products (red), wine (orange), human sources (green), meat products (brown), unknown sources (pink). Rows of the dendrogram show similarity relationships between strains based on phenolic compounds tolerance. Columns of the dendrogram show the similarity among the effect of phenolic compounds on *Lactiplantibacillus* strains.

In this study, only 19 strains (mostly *Lpb. pentosus* from olives) were able to grow in the presence of 100 mM gallic acid, while most strains (n. 73) grew in media supplemented with 50 mM protocatechuic acid and (n. 66) 25 mM syringic acid (Figure 1). Hydroxybenzoic acid had the highest toxicity, and none of the strains were able to grow at the highest concentration (40 mM). On the contrary, lower levels of phenolic acids (gallic ≤50 mM, protocatechuic ≤25 mM, hydroxybenzoic ≤20 mM and syringic ≤12.5 mM) did not inhibit the growth of strains. The analysis of the minimum inhibitory concentration (MIC) grouped the strains into three different clusters (Figure 2): the first one (C1) included strains with the lowest tolerance to phenolic compounds; the second large group (C2) included strains able to cope with the highest levels of protocatechuic (50 mM) and syringic (25 mM) acids, but unable to tolerate high concentrations of gallic (100 mM) and 4-hydroxybenzoic (40 mM) acids; the third cluster (C3) grouped the most tolerant lactobacilli (isolated from olives and sourdoughs), characterized by their greater resistance to gallic acid (100 mM).

As already demonstrated [12], we confirmed that the capability of the *Lactiplantibacillus* group to survive to olive-associated phenols is strain-specific; however, compared to other studies in which a limited number of strains has been tested, in this work, a relatively large collection of strains was considered, improving the information on the functional diversity of the *Lactiplantibacillus* group.

Oleuropein, the secoiridoid glycoside that causes the bitter taste of olives, is another important phenolic compound, and its degradation is essential to make olives and their derived products edible for humans. Oleuropein is hydrolysed into glucose and aglycone by *β*-glucosidases of both endogenous (olive fruit) or exogenous (microbial) origin. In turn, oleuropein aglycone is converted into elenolic acid and hydroxytyrosol (non-bitter compounds) through the activity of an esterase enzyme or, in part, through acidic hydrolysis. Ligstroside, which differs from oleuropein only because the tyrosol replaces the hydroxytyrosol moiety, undergoes a similar fate to oleuropein.

In this study, the capability to degrade oleuropein (TLC assay; Figure S1) was widespread among the strains (n. 71; *β*-glucosidase activity), while the capability to release hydroxytyrosol (esterase activity) was found only in 50 lactobacilli (Figure 1; Table S4). The source of isolation, however, did not affect oleuropein degradation and hydroxytyrosol formation, as these features were also found in strains isolated from matrices other than olives and brines (Figure 1). Similar to our results, Zago et al. [36] found that some *Lpb. plantarum* strains isolated from several cheeses were able to hydrolyse oleuropein. The hydrolysis of oleuropein has been studied for a long time in the *Lactiplantibacillus* group [6,7,12,25,46], and it is well known that this feature is strain-dependent.

During olive fermentation (especially for non-alkali treated fruits), *Lactiplantibacillus* starters may reduce the oleuropein content [47], debittering the final product and increasing the hydroxytyrosol level. Ramírez et al. [48] demonstrated that the degrading ability of *Lpb. plantarum* and *Lpb. pentosus* strains was significantly different, and only a few strains were able to hydrolyse most of the oleuropein (up to 90% of initial concentration) added to the reaction medium. This underlines that the debittering capability of *Lactiplantibacillus* strains (besides the presence of *β*-glucosidase and esterase activities) may also be affected by oleuropein content, which in turn is related to the olive cultivars [49]. More recently, Vaccalluzzo et al. [6] found that the oleuropeinolytic activity of *Lpb. plantarum* strains was affected by a low temperature, rather than high salt concentrations and low pH values.

Oleuropein, moreover, may affect the functionality of the starter cultures regulating the transcriptional levels of genes involved in the growth, transport, carbohydrate metabolism, bacteriocin production and quorum sensing of *Lpb. plantarum* [50]; therefore, the capability to degrade and use it is crucial for the selection of functional and competitive cultures.

The ability to form hydroxytyrosol is a desired feature among *Lactiplantibacillus* strains, as it may improve the antioxidant properties of olive-derived products, providing benefits for human health; free hydroxytyrosol, in fact, is one of the most powerful free radical scavengers, and it is also a metal chelator, exerting potential cardioprotective, anticancer, endocrine regulator and antimicrobial effects (in vitro and in vivo trials, however, demonstrated that the potential benefits are related to both dose and timing of intake [51,52]). To this purpose, EFSA indicated that the consumption of olive oil containing hydroxytyrosol or tyrosol as well as their derivatives (daily intake of 5 mg Hyt/20 g olive oil) helps to protect blood lipids from oxidative stress (Commission Regulation EU n.432/2012).

### 3.3. Characterization of 15 Selected Lactiplantibacillus Strains

The capability to scavenge radicals, to form biofilm, to grow in the presence of different NaCl concentrations and pH values, to survive in phenolic-rich matrices and to simulate the gastro-intestinal tract (GIT) was investigated in 15 selected strains (* in Figure 1 and Figure 2). The strains were chosen mainly on the basis of phenolic compound tolerance, but other features (e.g.*,* salt tolerance, oleuropein degradation and hydroxytyrosol formation, as well as EPS production) were also considered. *Lpb. plantarum* subsp. *plantarum* ISLCPT57, which lacked most of these important properties, was included for comparison.

**Figure 3:**
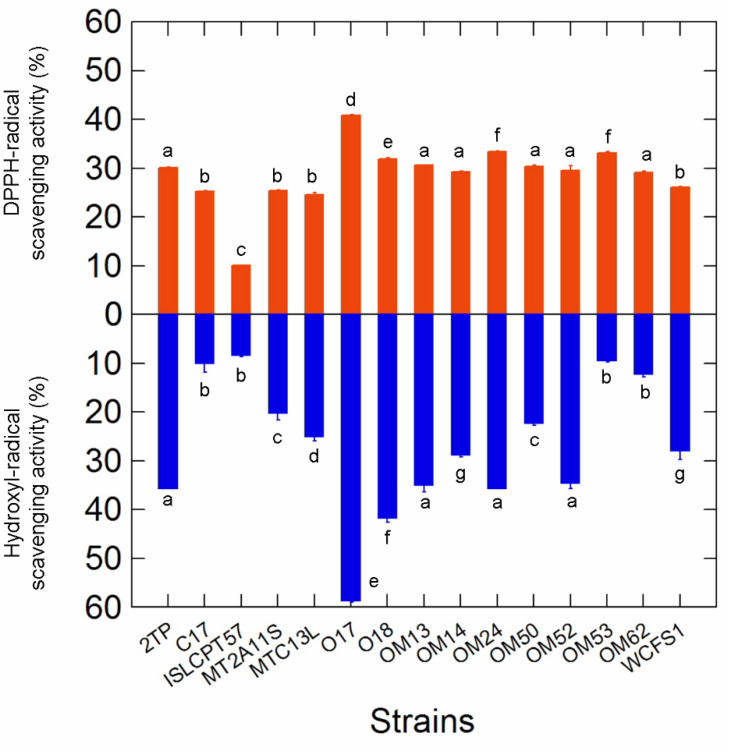
DPPH and hydroxyl radical scavenging activities in the 15 selected *Lactiplantibacillus* strains. Letters on plot bars indicate significant differences (Tukey*′*s HSD, *p* < 0.01) in scavenging activity within strains.

A wide variability among *Lactiplantibacillus* strains was observed in hydroxyl radical scavenging activity (ranging from 60% for *Lpb. pentosus* O17 to 8% for *Lpb. plantarum* subsp. *plantarum* ISLCPT57; Figure 3). On the contrary, although the capability to remove DPPH radicals ranged from 40% (*Lpb. pentosus* O17) to 10% (*Lpb. plantarum* subsp. *plantarum* ISLCPT57), most of the strains exhibited comparable detoxification activity. In both cases, *Lpb. pentosus* O17 exhibited the highest scavenging ability.

LAB are able to scavenge toxic radicals by producing different antioxidant enzymes (i.e., superoxide dismutase, catalase, flavin-dependent oxidases/peroxidases, thioredoxin and glutathione reductases). Radical scavenging activity may prevent oxidative processes in foods, ensuring the stability of proteins, lipids and phenolic compounds.

Kachouri et al. [53] demonstrated that a *Lpb. plantarum* strain with radical scavenging properties increased the phenolic content and total antioxidant activity of Chetoui olives during the storage period. The beneficial effect of antioxidant strains was evaluated also in other olive-derived products; some authors [54,55], for example, demonstrated that virgin olive oil produced with fruits inoculated with *Lpb. plantarum* had higher total phenolic content and greater stability to oxidative processes than that obtained from uninoculated drupes. The use of *Lpb. plantarum*, moreover, increased the antioxidant activity of OMW [56], reducing the auto-oxidation of phenolic compounds.

**Figure 4:**
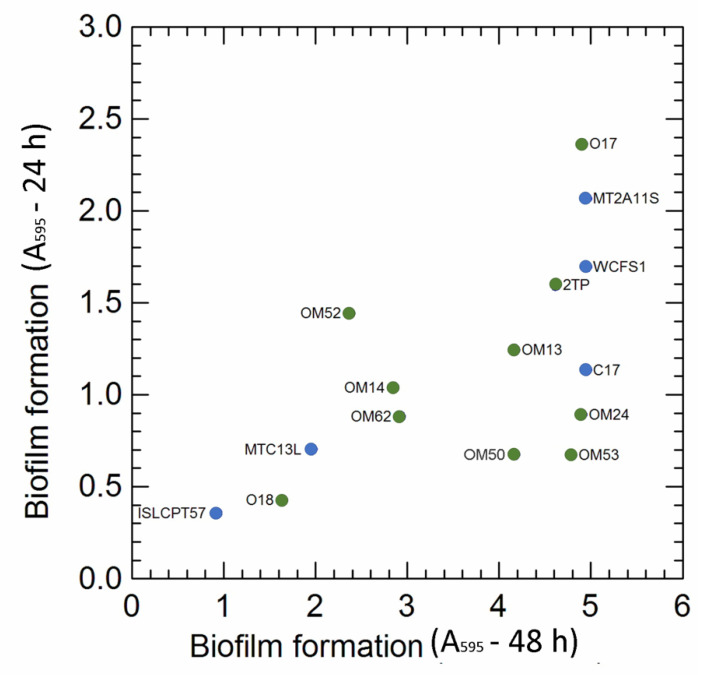
Biofilm formation in the 15 selected *Lactiplantibacillus* strains after 24 and 48 h incubation. Colour: green *Lpb. pentosus* strains (isolated from olive and brine); blue, *Lpb. plantarum* strains (isolated from other sources, e.g.*,* sourdoughs, cheese, human).

In this study, the potential capability to form stable biofilms (CV assay) was also evaluated. The A_595_ values (Figure 4) differed among the strains and, for most of them, were dependent on incubation time; prolonged cultivation (48 h), in fact, increased (by 1.6 and 7.1 times, respectively, for *Lpb. pentosus* OM52 and OM53) the fraction of adherent cells compared to 24 h of incubation. *Lpb. pentosus* O17 and *Lpb. plantarum* MT2A11S exhibited the highest ability in biofilm formation after both 24 and 48 h of growth, while *Lpb. plantarum* subsp. *plantarum* ISLCPT57 had the lowest fraction of adherent cells. In this study, the capability to form biofilm was not correlated to EPS production ability, since some of the best biofilm producers (e.g.*, Lpb. pentosus* O17, *Lpb. plantarum* MT2A1 and WCFS1) were unable to synthetize EPS from the sugars used in the screening step (see Table S3).

During the olive fermentation process, *Lactiplantibacillus* strains were able to shape a compact biofilm on both biotic (olives skin) and abiotic (glass slides) surfaces to ensure their survival over time [57,58,59,60]. Biofilm formation, however, depends on several factors, including the physicochemical interactions between bacterial cells, the properties of the olive surface and the aqueous phase, the capability to produce EPS and the presence of specific regulatory genes [58,59,60].

Several authors demonstrated that table olives supported the formation of poly-microbial biofilms, including a cooperation between LAB (*Lpb. plantarum* [43,57,58] and *Lpb. pentosus* [43,60,61]) and yeasts (*Candida famata*, *Candida ciferrii*, *Rhodotorula mucilaginosa*, *Crytococcus laurentii*, *Pichiaguillier mondii* [58], *Pichia galeiformis*, *Candida sorbosa*, *Geotrichum candidum* [57,61] and *Pichia membranifaciens* [60]).

Kachouri et al. [58], using a scanning electron microscope, demonstrated the ability of *Lpb. plantarum* to adhere to olive surfaces during the storage of Chetoui table olives; the biofilm, moreover, promoted a reduction of undesirable microorganisms (moulds and yeasts already present on olive fruits) probably due to competition for nutrients and oxygen, the synthesis of antimicrobial products or modifications in the physicochemical properties of the olives.

The ability to adhere to olive surfaces may be also a survival strategy during transit into gastro-intestinal tract (GIT). Some *Lactobacillus* strains may have functions beneficial to the human host and, thus, the ability to cope with GIT conditions may be a convenient selection criterion.

**Figure 5:**
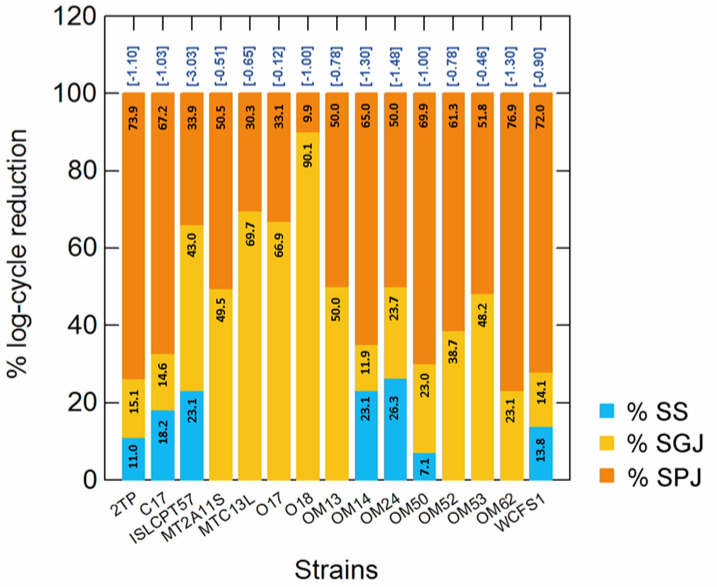
Percentage (%) of log cycle reduction measured at each step (numbers within bars) of simulated gastro-intestinal (GIT) transit (simulated saliva, SS; simulated gastric juice, SGJ; simulated pancreatic juice, SPJ). For each strain, survival was calculated as a reduction of log(N/N_0_), where N_0_ and N are the number of cells before and after exposure to each stress. The numbers in brackets, over the bars, indicate the overall log cycle reduction after all steps (complete simulated GIT transit).

In this study, the resistance to GIT-associated stresses was also evaluated. The % of log cycle reduction in simulated saliva (SS), gastric (SGJ) and pancreatic (SPJ) juices are shown in Figure 5. With the exception of *Lpb. plantarum* ISLCPT57, which was sensitive to simulated GIT transit (3.03 log cycle reduction), the other *Lactiplantibacillus* strains were quite tolerant, showing an overall decrease in survival ranging from 0.12 to 1.48 log cycles. As expected, SS conferred the least stress, while the effect of SGJ and SPJ was strain-dependent. *Lpb. pentosus* O17 was the most tolerant strain.

Arroyo-López et al. [62], using a TIM model to simulate gastric and intestinal human digestion, showed a considerable resistance of two olive-associated *Lpb. pentosus* strains to gastric digestion and the distal tract of the GIT (i.e.*,* the jejunum and ileum). Botta et al. [63] demonstrated several potential probiotic features of *Lpb. plantarum* and *Lpb. pentosus* strains, including the resistance to simulated gastric digestion (human intestinal epithelial cell line model). These studies underline the possible use of fermented table olives as a functional food, being a promising vehicle to transfer microorganisms into the human body [5,62,63].

During the production of fermented olives, both natural microbiota and starter cultures must survive in the presence of the high salt concentrations and low pH values that characterize most fermentation brines [5,10].

**Figure 6:**
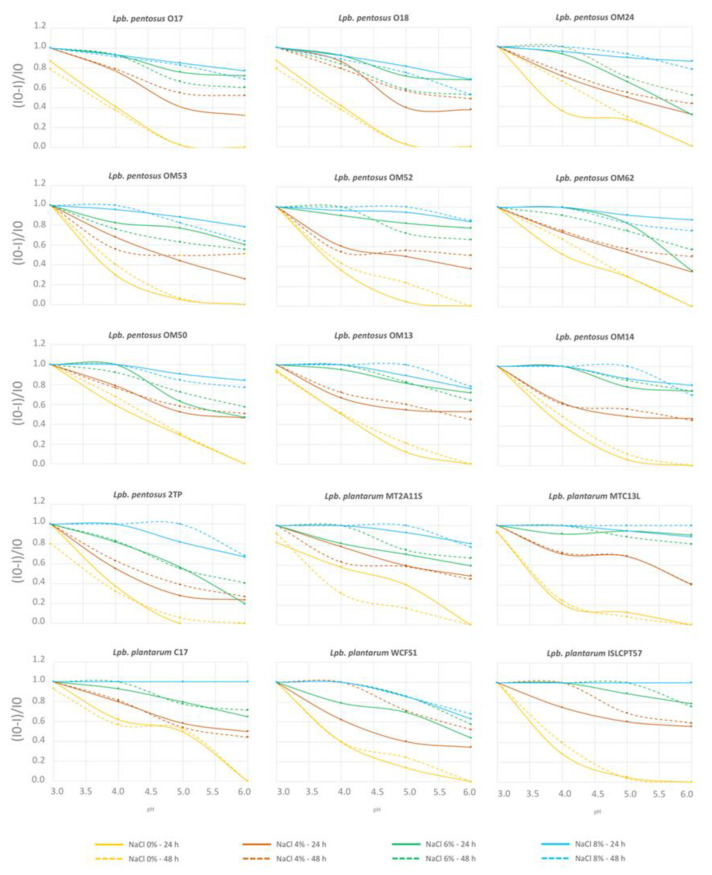
Inhibition curves in response to NaCl concentrations (blue, 8% NaCl; green, 6% NaCl; red, 4% NaCl; orange, 0% NaCl) and pH values (x-axis), after 24 h (continuous lines) and 48 h (dotted lines) of incubation. Inhibition was calculated as (I0-I)/I0, where I0 was the response of the control (i.e., A650 at 0% NaCl and pH 6.0) and I is the response at any given concentration of salt and pH value.

In this study, the ability to grow at different combinations of NaCl concentrations and pH values was evaluated using a synthetic medium. The harshest combination (8% NaCl, pH 3.0) inhibited the growth of all strains after both 24 and 48 h of incubation (Figure 6). Overall, salt concentrations ≥ 6% NaCl significantly impaired strain cultivation, regardless of the pH conditions. Survival in less severe combinations (i.e.*,* NaCl ≤ 6%; pH ≥ 4.0*–*4.5 commonly reached during olive fermentation), however, was also strain-specific.

### 3.4. Survival in Olive Mill Wastewater

As already mentioned, the resistance of *Lactiplantibacillus* to individual phenolic compounds has been reported for several strains [12], but the response to complex phenol-rich matrices (with the interaction of several components) has been scantly investigated. In this study, we used OMWs from *Leccino*, *Coratina* and *Cima di Melfi* cultivars as model substrates. OMW resulting from olive oil production, in fact, contains up to 80% of total phenols and fully reflects the phenolic profile of olive fruits.

In this study, no strain was able to survive in undiluted OMW, while tolerance to diluted OMWs (15 min incubation) depended on the cultivar (Table 2). *Coratina* affected strain survival to a greater extent, while *Cima di Melfi* provided the least stressful conditions. The most tolerant strains (*Lpb. pentosus* O17 and OM24 from olives, *Lpb. plantarum* C17 from cheese and *Lpb. plantarum* MTDA11S from sourdoughs) were further tested for OMW tolerance by using prolonged incubation times (Figure S2). The results confirmed that the *Cima di Melfi* OMW had the lowest inhibitory activity, while the survival in *Coratina* and *Leccino* OMWs depended on both strain and incubation time. *Lpb. pentosus* O17 exhibited the greatest robustness to OMWs, with all samples coping even for 30 min (albeit at very diluted concentrations).

**Figure 7:**
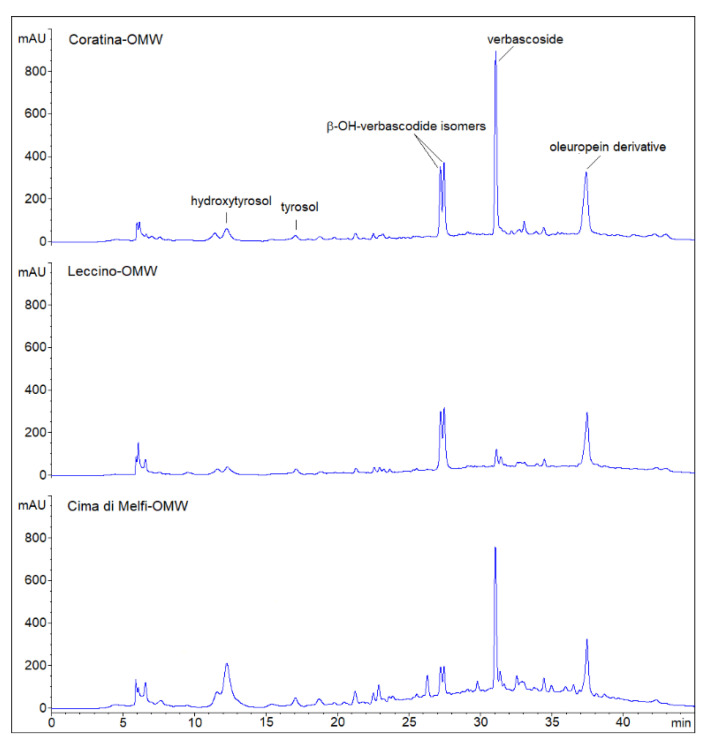
Phenolic profiles (HPLC chromatograms) of olive mill wastewater (OMW) resulting from olive oil production of *Leccino*, *Coratina* and *Cima di Melfi* cultivars. Peaks have been putatively assigned on the basis of retention time values (min) and by comparing them with literature data and UV spectra (280 nm).

To understand the possible factors affecting strain survival in OMWs, the total polyphenol content (TPC) and the phenolic profile were evaluated for all OMW samples. *Cima di Melfi* had the highest TPC (11.9 ± 0.3 g/kg) compared to the *Coratina* OMW (8.8 ± 0.2 g/kg) and *Leccino* (7.2 ± 0.3 g/kg) cultivars. Verbascoside, β-OH-verbascoside isomers and an oleuropein derivative were the main phenolic compounds of OMWs (Figure 7), although their occurrence was cultivar-dependent. Verbascoside was abundant in *Coratina* and *Cima di Melfi* samples, but was hardly present in the *Leccin*o cultivar; β-OH-verbascoside, instead, was significantly present in *Coratina* and *Leccin*o OMWs, but not in *Cima di Melfi*. An oleuropein derivative was common to all cultivars at comparable concentrations. The content of tyrosol and hydroxytyrosol was low in all samples. Overall, *Coratina* OMW had the highest amounts of all detected phenolic compounds and, most likely, this was correlated to its stronger inhibitory activity.

The antimicrobial activity of OMW and the synergistic effect of related phenolic compounds have been demonstrated by several authors. Tafesh et al. [64] found that some Gram+ (e.g., *Streptococcus pyogenes* and *Staphylococcus aureus*) and Gram− (e.g., *Escherichia coli* and *Klebsiella pneumoniae*) pathogens may be inhibited by using OMW extracts. Abu-Lafi et al. [65] also demonstrated that OMW extracts had an inhibitory activity against *St. aureus* and *E. coli* that was higher than that of antibiotics commonly used for their inactivation.

More recently, Belaqziz et al. [66] suggested that the antioxidant and antimicrobial activity (i.e., against *Lactococcus lactis*, *Lactobacillus bulgaricus*, *St. aureus*, *Bacillus subtilis*, *L. innocua*, *Escherichia coli* and *Salmonella typhimurium*) of OMW and table olive wastewaters (TOW) depended on both total phenolic content and the relative balance of phenolic compounds.

In this study, *Lactiplantibacillus* strains significantly suffered in OMW, and most of them survived when incubated in diluted samples (from 25% to 3.12%; from 4 to 32 dilutions of TPC of each cultivar). Previously, Ayed and Hamdi [67] demonstrated that *Lpb. plantarum* was able to survive in diluted OMW, removing 46% of total phenolic compounds; successively, Kachouri et al. [56] confirmed that undiluted OMW inhibited the growth of *Lpb. plantarum*, but in diluted samples, the strain led to degradation and removal (up to 63%) of several phenolic compounds (e.g., tyrosol, gallic, *p*-coumaric and caffeic acids).

In this study, the presence and interaction of verbascoside and β-OH-verbascoside isomers (found in *Coratina* cv.) mainly affected the strain survival. The high amounts of verbascoside and β-OH-verbascoside isomers in *Coratina* OMW was already demonstrated by Cardinali et al. [30] and D′Antuono et al. [68]; phenylpropanoid glycoside, however, was also found in OMW, resulting from different cultivars of Greek olives (e.g., Koroneiki and Lianiolia [68,69]).

### 3.5. Genome Sequencing of Lpb. pentosus O17 and Occurrence Analysis of Genes Involved in Degradation and Metabolism of Phenolic Compounds

The draft genome of *Lpb. pentosus* O17 (average coverage of 800.0×) contained 53 contigs, a circular chromosome of 3,850,701 bp and an overall GC content of 45.9%. A total of 3401 protein-coding sequences, 54 pseudogenes, 59 tRNA genes, 3 rRNA genes, 4 noncoding RNA (ncRNA) and 5 CRISPR arrays were identified.

The occurrence of the most important genes (*β*-glucosidase, gallate decarboxylase subunits B, C and D, *p*-coumaric acid decarboxylase, esterase, carboxylesterase, tannase subunits A and B and transcriptional regulator *PadR*) involved in the degradation and metabolism of phenolic compounds (Table S2; published data available for *Lpb. plantarum* and *Lpb. pentosus* strains) was verified in the genome of *Lpb. pentosus* O17.

The strain has an interesting gene pool and lacks only carboxylesterase and gallate decarboxylase subunits B and D. The gene profile of *Lpb. pentosus* O17 was compared with that of 28 (4 finished, 24 permanent draft) publicly available *Lpb. pentosus* genomes.

The genes of *β*-glucosidase, esterase, gallate decarboxylase (subunits B and C; *lpdB* and *lpdC*), *p*-coumaric acid decarboxylase (except for *Lpb. pentosus* 1.8.9), carboxylesterase and the transcriptional regulator *PadR* were present in all *Lpb. pentosus* genomes analysed (Table S5). On the contrary, the occurrence of gallate decarboxylase (subunit D; *lpdD*) and tannase (subunits A and B; *tanA* and *tanB*) sequences slightly differed among the analysed genomes; *tanA* and/or *tanB* occurred in most of them (93%), while *lpdD* was present only in *Lpb. pentosus* 3.2.8. Most of the strains were isolated from vegetable materials (i.e., corn silage, cucumber or olive fermentation and mustard pickles), while two strains were collected from human (vagina of a healthy Nigerian woman) and environmental (temperate deciduous forest biome soil) sources.

Most of the studies related to the genes involved in the metabolism of phenolic compounds focused on *Lpb. plantarum* species, although some data were also present for *Lpb. paraplantarum* and *Lpb. pentosus* species.

The metabolism of phenolic compounds is dynamic and quite complex. Besides the presence of genes, it is crucial to understand the interaction and regulation mechanisms of related degrading enzymes. Carrasco et al. [17] found that in some *Lpb. pentosus* strains, the exposure to different phenolic compounds (i.e., gallic, *p*-coumaric, caffeic, ferulic, vanillic, oleuropein, tyrosol, hydroxytyrosol and verbascoside) induced the concurrent over-expression of several genes (i.e., gallate decarboxylase, *p*-coumaric decarboxylase and tannase), suggesting that the degradation and metabolism of phenolic compounds may be related to the synergic effect of different degrading enzymes.

Landete et al. [12] reported that, in *Lpb. plantarum*, gallate decarboxylase may be involved in the decarboxylation of both gallic acid and protocatechuic acid, and tannase (a specific class of esterase) may also exert this function [70]. Actually, it was demonstrated that the sequential action of esterase and decarboxylases drives the proper degradation of hydroxybenzoic (tannase/gallate decarboxylase) and hydroxycinnamic (esterase/phenolic acid decarboxylase) acids [18]. Tannase was investigated in several strains of *Lpb*. *plantarum* but, to date, very few data [17,71] are available for *Lpb*. *paraplantarum* and *Lpb*. *pentosus* species. In this study, we found that tannase-encoding genes (mainly *tanB*) were widespread among *Lpb*. *pentosus* strains.

Although the *β*-glucosidase gene was present in all *Lpb*. *pentosus* genomes, phenotypic evidence demonstrated that its occurrence is not always related to oleuropein degradation [6,17] and, therefore, further studies are needed to correlate genetic information and strain metabolism.

## 4. Conclusions

A high level of diversity within the strains and species of the *Lactiplantibacillus* group was found in this study. In the screening step, the features that provided high variability among the strains were related to the capability to inhibit *Yarrowia lipolytica*, to produce EPS from glucose, maltose and sucrose, acid from xylose, hydroxytyrosol from oleuropein and to cope with gallic acid. On the contrary, most of strains tolerated protocatechuic and syringic acids, while hydroxybenzoic acid provided the highest level of toxicity.

Some selected strains were also able to produce biofilm, scavenge toxic radicals and survive the simulated GIT and simulated brine, suggesting their possible used as starter for the production of fermented table olives.

Moreover, although most of the strains had poor resistance to OMWs (used as a complex phenolic-rich matrix), our results provided progress in knowledge on the survival of the *Lactiplantibacillus* group to phenolic compounds. As the latter significantly differ (in terms of content and profile) among olive cultivars, the presence of proper degrading enzymes may be a helpful criterion for the selection of robust strains.

Additionally, the genome in silico analysis provided further information on the occurrence of genes involved in phenolic compound metabolism in *Lpb. pentosus* species, although the number of available genomes is significant lower compared to the closely related species *Lpb. plantarum*.

Contrary to some data available in the literature, in this study, some features related to the production of fermented olives (e.g., oleuropein degradation and hydroxytyrosol formation, tolerance to phenolic compounds and biofilm formation) were not strictly associated with isolation sources, and our data suggest that strains isolated from other sources (e.g., sourdoughs) can be also used as starter for the production of fermented table olives.

In this study, in fact, besides the olive-associated strain *Lpb. pentosus* O17, the *Lpb. plantarum* MT2A11S isolated from sourdoughs (both tolerant to phenolic compounds, able to produce biofilm and with high stress robustness) may also be used for the production of fermented olives.

## Figures and Tables

**Table 1:** List of *Lactiplantibacillus* strains used in this study.

Source Group	Source	Strains and Species
Cheese (CH)	Caciocavallo cheese	*Lpb. paraplantarum* F10, B7N26; *Lpb. plantarum* B7N23; *Lpb. plantarum* subsp. *plantarum* B15, C17, S12
	Cheese	*Lpb. plantarum* ISLCPT68; *Lpb. plantarum* subsp. *argentoratensis* FSL170; *Lpb. plantarum* subsp. *plantarum* ISLCPT57, FSM17
Meat product (M)	Salami	*Lpb. pentosus* LPL
Plant material (P)	Vegetables	*Lpb. plantarum* subsp. *plantarum* S85, LM3
	Silage	*Lpb. plantarum* subsp. *plantarum* DCU101, NCFB340
Olives (O)	Olive brine	*Lpb. pentosus* 2TP, 4TP, 4TG, 5TP, P13.3; *Lpb. plantarum* subsp. *plantarum* P1.5
	Treated table olives (*Cerignola* cv.)	*Lpb. pentosus* O5 *; *Lpb. plantarum*: O1 *, O4 *
	Natural table olives (*Cerignola* cv.)	*Lpb. pentosus* O11 *, O12 *, O15 *; *Lpb. plantarum* O13*
	Brine from treated table olives (*Cerignola* cv.)	*Lpb. pentosus* O17 *, O18 *, O19 *, O20 *
	Brine from natural table olives (*Cerignola* cv.)	*Lpb. pentosus* O24 *
	Table olives (*Nocellara del Belice* cv.)	*Lpb. pentosus* OM24, OM53, OM52, OM62, OM50, OM13, OM14, OM35 ^#^
Sourdough (SD)	Manioca	*Lpb. plantarum* subsp. *plantarum* DKO22, 38AA
	Tapioca	*Lpb. plantarum* subsp. *argentoratensis* DK36
	Fermented millet	*Lpb. plantarum* subsp. *argentoratensis* CNRZ1890
	Ogi	*Lpb. plantarum* subsp. *argentoratensis* NCIMB12120
	Altamura bread	*Lpb. plantarum* PA20S, PE2S
	Carasau bread	*Lpb. plantarum* subsp. *plantarum* 1069, 872
	Cornetto di Matera bread	*Lpb. paraplantarum* MTG8L, MTG30L; *Lpb. plantarum* MT2A11S, MT2D3S; *Lpb. plantarum* subsp. *argentoratensis* MTC13L; *Lpb. plantarum* subsp. *plantarum*: MTD12L, MT2D20S, MTNTA3S, MT2D6S, MT2D7S, MT2D25L, MT2S, MTF13S, MTF1L, MTF28L, MTF9L
	Moddizzosu bread	*Lpb. plantarum* subsp. *plantarum* 954
	Spianata bread	*Lpb. plantarum* subsp. *plantarum* 1089
	Zichi bread	*Lpb. plantarum* subsp. *plantarum* 1505, 1513
Wine (W)	Wine	*Lpb. plantarum* B161
		*Lpb. plantarum* subsp. *plantarum* UT2.1, US3.1, UBS3
Human (H)	Human	*Lpb. plantarum* WCFS1
	Saliva	*Lpb. plantarum* subsp. *plantarum* NCIMB8826
Unknown (U)	Unknown	*Lpb. plantarum* subsp. *plantarum* S2A19LPa

* Strains isolated and identified in this study. # Strains from the Microbial Culture Collection of the Department of Food Science, University of Naples Federico II, Portici, Italy. The other strains belong to the Microbial Culture Collection of Industrial Microbiology Laboratory, University of Basilicata, Potenza, Italy.

**Table 2:** Strain survival after exposure (15 min) to different concentrations of olive mill wastewater (OMW) derived from olive oil production of *Leccino*, *Coratina* and *Cima di Melfi* cultivars.

Strains	Species	Dose Causing ≥ 7 log Reductions ^a^		
		OMW ^b^ *Leccino*	OMW ^b^ *Coratina*	OMW ^b^ *Cima di Melfi*
O17	*Lpb. pentosus*	50	25	50
O18	*Lpb. pentosus*	25	12.5	50
OM24	*Lpb. pentosus*	12.5	12.5	50
OM53	*Lpb. pentosus*	25	6.25	50
OM52	*Lpb. pentosus*	6.25	6.25	25
OM62	*Lpb. pentosus*	6.25	6.25	25
OM50	*Lpb. pentosus*	6.25	6.25	25
OM13	*Lpb. pentosus*	12.5	6.25	25
OM14	*Lpb. pentosus*	6.25	12.5	25
2TP	*Lpb. pentosus*	12.5	6.25	25
MT2A11S	*Lpb. plantarum*	25	25	25
WCFS1	*Lpb. plantarum*	12.5	6.25	25
C17	*Lpb. plantarum* subsp. *plantarum*	12.5	25	25
ISLCPT57	*Lpb. plantarum* subsp. *plantarum*	6.25	3.12	12.5
MTC13L	*Lpb. plantarum* subsp. *argentoratensis*	12.5	6.25	12.5

^a^ The concentration of cells used to inoculate the different percentage (%) of OMW was 7 log cfu/mL. ^b^ OMW was diluted with NaCl 0.85% (*w*/*v*); the % of OMW corresponds to the following dilution (d): OMW 50% = 2d; OMW 25% = 4d; OMW 12.5% = 8d; OMW 6.25% = 16d; OMW 3.12% = 32d.

## Data Availability

Not applicable.

## Supplementary Materials

The following supporting information can be downloaded at: https://www.mdpi.com/article/10.3390/microorganisms10030625/s1, Table S1: Reaction mixture and PCR program for the amplification of partial *16S rRNA* genes; Table S2: List of genes involved in phenolic compound metabolism, used to verify the occurrence in publicly available genomes of *Lactiplantibacillus paraplantarum, Lpb. plantarum* and *Lpb. pentosus* (References [72,73,74,75] are cited in the Table S2); Table S3: Exopolysaccharide (EPS) production and antimicrobial activity of strains; Figure S1: Thin layer chromatography (TLC) of MRS samples unsupplemented and supplemented with oleuropein and hydroxytyrosol; Table S4: Qualitative evaluation of oleuropein degradation and hydroxytyrosol formation using thin layer chromatography (TLC); Figure S2: Resistance of *Lpb. pentosus* O17, OM24 and *Lpb. plantarum* C17, MT2A11S to different concentrations (from 50% to 6.12% *v*/*v*) of *Leccino*, *Coratina* and *Cima di Melfi* OMWs after 15, 30, 60 and 120 min of incubation at 30 °C; Table S5: Occurrence analysis of genes involved in degradation and metabolism of phenolic compounds in *Lactiplantibacillus pentosus* genomes.
