# Supplementary material for: Selection of Lactiplantibacillus Strains for the Production of Fermented Table Olives
Source: Microorganisms. 2022 Mar 15;10(3):625. doi: 10.3390/microorganisms10030625 (PMC8956003; doi:10.3390/microorganisms10030625)
Supplement: Supplementary file 1 [file microorganisms-10-00625-s001.zip › microorganisms-1632959-supplementary material.pdf]

Title

Selection of *Lactiplantibacillus* strains for the production of fermented table olives

Authors

Teresa Zotta [1], Marilisa Giavalisco [1], Eugenio Parente [1,*], Gianluca Picariello [2], Francesco Siano [2], Annamaria Ricciardi [1]

Affiliations

[1] Scuola di Scienze Agrarie, Alimentari, Forestali ed Ambientali (SAFE), Università degli Studi della Basilicata, 85100 Potenza, Italy; [2] Istituto di Scienze dell'Alimentazione-CNR, 83100 Avellino, Italy

* Corresponding author

Prof. Eugenio Parente

E-mail: eugenio.parente@unibas.it Tel.: +39-0971-205561

Table S1. Reaction mixture and PCR-program for the amplification of partial 16S rRNA gene.

**Reagents**	**Reaction mixture concentration (50 μL)**	**Amplification program ***
Deionized water	-	Initial denaturation: 95°C for 5 minutes
Reaction buffer (WonderTaq, Euroclone)	1x	
Primer forward 27F-ND	0.5 μM	30 cycle of: denaturation (95°C, 45 sec.), annealing (54°C, 45 seconds) and extension (72°C, 45 seconds)
Primer reverse 1492R-ND-L	0.5 μM	
Taq (WonderTaq, Euroclone)	0.025U/μL	
DNA	50 ng/μL	Final extension: 72°C for 7 minutes

* PCR was carried out in a T100™ Thermal Cycler Bio-Rad (Bio-Rad Laboratories Srl, Segrate, Milan, Italy); PCR products (1535 bp) were separated (90 min at 100 V) on 1.5% w/v agarose gel, stained with 0.05 μL/mL of GelRed™ (10,000*x* in water; Botium Inc., Fremont, California) and visualized using GelDoc XR system (Bio-Rad Laboratories).

Table S2. List of genes involved in phenolic compound metabolism, used to verify the occurrence in publicly available genomes of *Lactiplantibacillus paraplantarum*, *Lpb. plantarum* and *Lpb. pentosus*.

Gene annotation	COG categories*	Strain	Accession n.	Locus tag (symbol)	Size (bp/aa)	References
β-glucosidase/6-phospho-β-glucosidase	G (COG 2723)	*Lpb. plantarum* WCFS1	CCC78348	lp_0906 (pbg2)	1503 bp/500 aa	[1]
aromatic acid carboxylase, subunit B	H (COG 0163)	*Lpb. plantarum* WCFS1	CCC77798	lp_0271 (*lpdB*)	564 bp/187 aa	[1, 2]
3-octaprenyl-4-hydroxybenzoate carboxy-lyase, UbiD family	H (COG 0043)	*Lpb. plantarum* WCFS1	CCC80016	lp_2945 (*lpdC*)	1473 bp/490 aa	[1-3]
aromatic acid carboxylase, subunit D	-	*Lpb. plantarum* WCFS1	CCC77799	lp_0272 (*lpdD*)	411 bp/136 aa	[1, 2]
phenolic acid decarboxylase	Q (COG 3479)	*Lpb. plantarum WCFS1*	CCC80619	lp_3665 (*padA*)	537 bp/178 aa	[1, 4]
transcriptional regulator of phenolic acid metabolism, PadR family	K (COG 1695)	*Lpb. plantarum* WCFS1	CCC80618	lp_3664 (*padR*)	546 bp/181 aa	[1]
tannase (tannin acyl hydrolase)	I (COG 0657)	*Lpb. plantarum* WCFS1	CCC80022	lp_2956 (*tanLp1*, $tanB_{Lp}$)	1410 bp/469 aa	[1-3, 5, 6]
hypothetical protein (with tannase-like activity)	-	*Lpb. plantarum* ATCC 14917	KRL35904	HMPREF0531_11477 ($tanA_{Lp}$)	1881 bp/626 aa	[1, 6-8]
carboxylesterase	Q (COG 1647)	*Lpb. plantarum* WCFS1	CCC78257	lp_0796	750 bp/249 aa	[5]
esterase	I (COG 0657)	*Lpb. plantarum* WCFS1	CCC80020	lp_2953	750 bp/249 aa	[2]
alpha/beta hydrolase	I (COG 0657)	*Lpb. plantarum* JDM1	WP_015825406	JDM1_1092	885 bp/295 aa	[6, 9]

* G: carbohydrate transport and metabolism; H: coenzyme transport and metabolism; Q: secondary metabolites biosynthesis, transport and catabolism; K: transcription; I; Lipid transport and metabolism. In bracket COG family.

Table S3. Exopolysaccharides (EPS) production and antimicrobial activity of strains.

EPS production				Antimicrobial activity	
**Strains**	**G-MRS [a]**	**M-MRS [a]**	**S-MRS [a]**	***Y. lipolytica* [b]**	**halo [c]**
B15 (Lpl)	+	+	+	CNRZ1890 (Lpl)	23.0
38AA (Lpl)	+	+	+	WCFS1 (Lpl)	18.1
B7N23 (Lpl)	+	+	+	MT2D6S (Lpl)	18.1
MTD12L (Lpl)	+	+	+	MT2D7S (Lpl)	17.9
MTNTA3S (Lpl)	+	+	+	MTC13L (Lpl)	17.2
MT2D6S (Lpl)	+	+	+	1513 (Lpl)	13.1
MT2D7S (Lpl)	+	+	+	872 (Lpl)	13.4
MTC13L (Lpl)	+	+	+	4TP (Lpe)	19.0
MT2S (Lpl)	+	+	+	954 (Lpl)	20.3
MTF13S (Lpl)	+	+	+	4TG (Lpe)	15.0
MTF1L (Lpl)	+	+	+	O4 (Lpl)	8.6
MTF28L (Lpl)	+	+	+	O19 (Lpe)	12.1
MTF9L (Lpl)	+	+	+	O20 (Lpe)	14.5
2TP (Lpe)	+	+	+	OM53 (Lpe)	11.6
5TP (Lpe)	+	+	+	OM52 (Lpe)	10.5
O4 (Lpl)	+	+	+		
O18 (Lpe)	-	+	-		
O19 (Lple)	+	+	+		
OM50 (Lpe)	-	-	+		
OM53 (Lpe)	-	-	+		

[a] G-MRS, M-MRS, S-MRS: MRS with 20 g/L maltose (M-MRS), or 20 g/L glucose (G-MRS), or 50 g/L sucrose (S-MRS) as carbon source (+, EPS-positive strains; -, EPS-negative strains); [b] *Y. lipolytica:* inhibitory activity of strains against *Yarrowia lipolytica* YL-12 (Deferred Antagonism Assay [10]); [c] halo: the size of inhibition zone (mm) was measured with a caliper. In brackets: Lpl, *Lpb. plantarum*; Lpe, *Lpb. pentosus.*

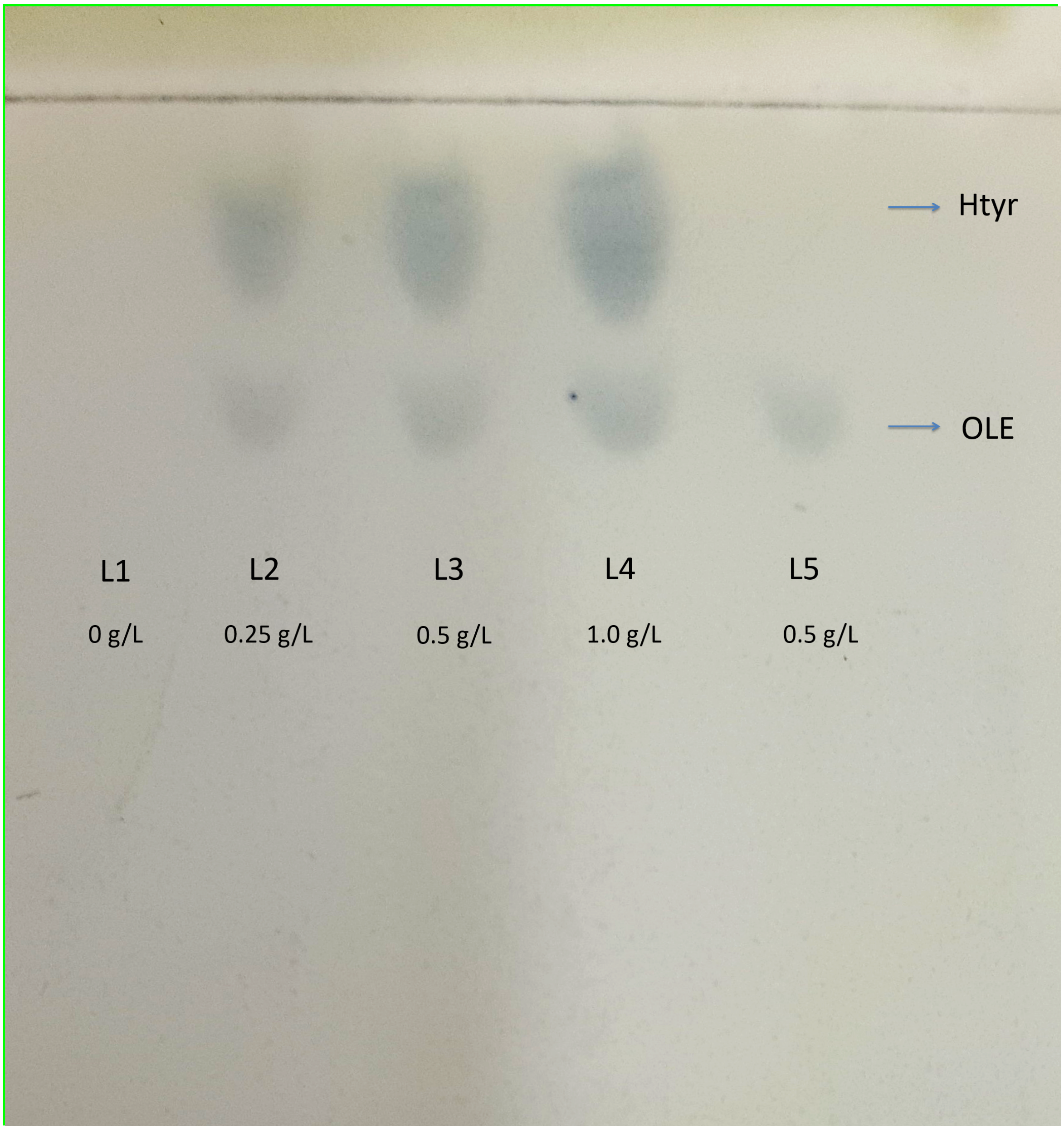

Figure S1. Thin Layer Chromatography (TLC) of MRS samples ([11]; *section 2.3*) unsupplemented (lane 1, L1) and supplemented with 0.25 g/L of oleuropein (OLE) and hydroxytyrosol (Htyr; L2), or with 0.5 g/L of OLE and Htyr (L3) or with 1 g/L of OLE and Htyr (L4) or only with 0.5 g/L of OLE (L5).

Table S4. Qualitative evaluation of oleuropein degradation and hydroxytyrosol formation using Thin Layer Chromatography (TLC).

**Strains**	**Oleuropein (OLE) TLC spot**		**Hydroxytyrosol (Htyr) TLC spot**	
	presence	absence	presence	absence
<u>*Lpb. paraplantarum*</u> B7N26<br><u>*Lpb. plantarum*</u> WCFS1, ISLCPT68, PA20S, PE2S, B7N23, MT2A11S, B161, O1, O13; *Lpb. plantarum* subsp. *plantarum* B15, C17, 1069, DKO22, S85, UBS3, P1.5, MTNTA3S, DCU101, 38AA, FSM170, S12, MT2D6S, 954, UT2.1, US3.1, S2A19LPa; *Lpb. plantarum* subsp. *argentoratensis* CNRZ1890, DK36, FSL170, MTC13L<br><u>*Lpb. pentosus*</u> 2TP, 4TP, 4TG, 5TP, P13.3, OM24, OM53, OM52, OM62, OM50, OM13, OM14, OM35, O12, O17, O18, O19, O20, O24	OLE degradation ability		Htyr formation ability	
<u>*Lpb. paraplantarum*</u> MTG30L, MTG8L<br><u>*Lpb. plantarum*</u> MT2D3S, O4; *Lpb. plantarum* subsp. p*lantarum* NCIMB8826, MTD12L, MT2D20S, ISLCPT57, MT2D7S, MT2D25L, MT2S, MTF13S, MTF1L, MTF28L, MTF9L, LM3; *Lpb. plantarum* subsp. *argentoratensis* NCIMB12120<br><u>*Lpb. pentosus*</u> LPL, O5, O11, O15	OLE degradation ability			Htyr formation inability
<u>*Lpb. paraplantarum*</u> F10<br><u>*Lpb. plantarum*</u> subsp. *plantarum* 1505, 1513, 872, 1089, NCFB340		OLE degradation inability		Htyr formation inability

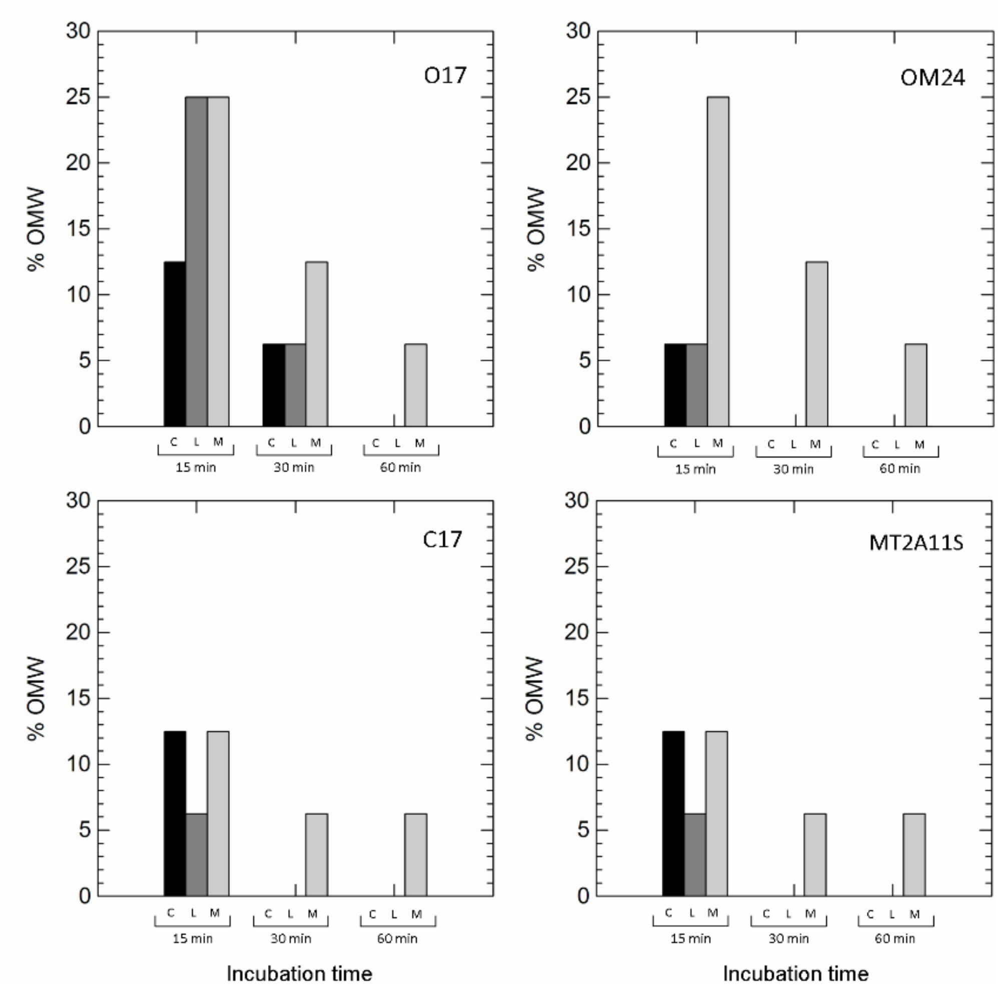

Figure S2. Survival of *Lpb. pentosus* O17, OM24 and *Lpb. plantarum* C17, MT2A11S to different concentrations (from 50% to 6.12% v/v) of *Leccino*, *Coratina* and *Cima di Melfi* OMWs after 15, 30, 60 and 120 minutes of incubation at 30°C.

Table S5. Occurrence analysis of genes involved in degradation and metabolism of phenolic compounds in *Lactiplantibacillus pentosus* genomes.

<table>
<tr><th>Strains</th><th>Sequencing status</th><th>Isolation source</th><th>pbg [a]</th><th>padA [b]</th><th>padR [c]</th><th>Carboxyl-esterase</th><th>lpdB [d]</th><th>lpdC [e]</th><th>lpdD [f]</th><th>Esterase</th><th>tanA [g]</th><th>tanB [h]</th></tr>
<tr><td>Lpb. pentosus 1.2.11, 1.2.13, 1.2.7, 1.8.18, 1.8.6, 14.2.16, 14.2.3, 14.8.42, 3.2.36, 3.2.37, 3.8.24, 3.8.45, 7.2.11, 7.2.15, 7.2.20, 7.2.23, 7.8.2, 7.8.46, LA0445, MU0445</td><td>Permanent draft</td><td>Cucumber fermentation</td><td rowspan="4">1</td><td rowspan="4">1</td><td rowspan="4">1</td><td rowspan="4">1</td><td rowspan="4">1</td><td rowspan="4">1</td><td rowspan="4">0</td><td rowspan="4">1</td><td rowspan="4">1</td><td rowspan="4">1</td></tr>
<tr><td>Lpb. pentosus ZFM222</td><td>Finished</td><td>Fermented vegetables</td></tr>
<tr><td>Lpb. pentosus SLC13</td><td>Finished</td><td>Mustard pickles</td></tr>
<tr><td>Lpb. pentosus BGM48</td><td>Finished</td><td>Olive fermentation</td></tr>
<tr><td>Lpb. pentosus 3.2.8</td><td>Permanent draft</td><td>Cucumber fermentation</td><td>1</td><td>1</td><td>1</td><td>1</td><td>1</td><td>1</td><td>1</td><td>1</td><td>1</td><td>1</td></tr>
<tr><td>Lpb. pentosus 1.8.9</td><td>Permanent Draft</td><td>Cucumber fermentation</td><td>1</td><td>0</td><td>1</td><td>1</td><td>1</td><td>1</td><td>0</td><td>1</td><td>1</td><td>1</td></tr>
<tr><td>Lpb. pentosus DSM 20314</td><td>Finished</td><td>Corn silage</td><td>1</td><td>1</td><td>1</td><td>1</td><td>1</td><td>1</td><td>0</td><td>1</td><td>1</td><td>0</td></tr>
<tr><td>Lpb. pentosus FL0421</td><td>Permanent draft</td><td>Temperate deciduous forest biome soil</td><td>1</td><td>1</td><td>1</td><td>1</td><td>1</td><td>1</td><td>0</td><td>1</td><td>0</td><td>0</td></tr>
<tr><td>Lpb. pentosus KCA1</td><td>Permanent draft</td><td>Vagina of a healthy Nigerian woman</td><td>1</td><td>1</td><td>1</td><td>1</td><td>1</td><td>1</td><td>0</td><td>1</td><td>0</td><td>1</td></tr>
<tr><td>Lpb. pentosus O17</td><td>Draft (this study)</td><td>Brine from treated table olives (Cerignola cv.)</td><td>1</td><td>1</td><td>1</td><td>0</td><td>0</td><td>1</td><td>0</td><td>1</td><td>1</td><td>1</td></tr>
</table>

[a] *pbg: β*-glucosidase; [b] padA: p-coumaric acid decarboxylase; [c] *padR*: Transcriptional regulator PadR; [d] *lpdB*: Gallate decarboxylase subunit B; [e] *lpdC*: Gallate decarboxylase subunit C; [f] *lpdD*: Gallate decarboxylase subunit D; [g] *tanA*: Tannase subunit A; [h] *tanB*: Tannase subunit B. 1: gene presence; 0: gene absence.

References

1. Carrasco, J.A.; Lucena-Padrós, H.; Brenes, M.; Ruiz-Barba, J.L. Expression of genes involved in metabolism of phenolic compounds by *Lactobacillus pentosus* and its relevance for table-olive fermentations. *Food Microbiol*. **2018**, *76*: 382–389. https://doi.org/10.1016/j.fm.2018.06.020.
2. Reverón, I.; Jiménez, N.; Curiel, J.A.; Peñas, E.; López de Felipe, F.; de las Rivas, B.; Muñoz, R. Differential gene expression by *Lactobacillus plantarum* WCFS1 in response to phenolic compounds reveals new genes involved in tannin degradation. *Appl. Environ. Microbiol*. **2017**, *83*, e03387-16. https://doi.org/10.1128/AEM.03387-16.
3. Jiménez, N.; Curiel, J.A.; Reverón, I.; de las Rivas, B.; Muñoz, R. Uncovering the *Lactobacillus plantarum* WCFS1 Gallate Decarboxylase Involved in Tannin Degradation. *Appl. Environ. Microbiol.* **2013**, *79*, 4253–4263. https://doi.org/10.1128/AEM.00840-13.
4. Rodriguez, H.; Landete, J.M.; Curiel, J.A.; de las Rivas, B.; Mancheño, J.M.; Muñoz, R. Characterization of the *p*-Coumaric acid decarboxylase from *Lactobacillus plantarum* CECT 748^T. *J. Agric. Food Chem*. **2008**, *56*, 3068–3072. https://doi.org/10.1021/jf703779s.
5. Esteban-Torres. M.; Reverón, I.; Mancheño, J.M.; de las Rivas, B.; Muñoz, R. Characterization of a Feruloyl Esterase from *Lactobacillus plantarum*. *Appl. Environ. Microbiol.* **2013**, *79*, 5130–5136. https://doi.org/10.1128/AEM.01523-13.
6. Landete, J.M.; Plaza-Vinuesa, L.; Montenegro, C.; Santamaría, L.; Reverón, I.; de las Rivas, B.; Muñoz, R. The use of *Lactobacillus plantarum* esterase genes: A biotechnological strategy to increase the bioavailability of dietary phenolic compounds in lactic acid bacteria. *Int. J. Food Sci. Nutr*. **2021b**, *72*, 1035–1045. https://doi.org/10.1080/09637486.2021.1900078.
7. Jiménez, N.; Esteban-Torres, M.; Mancheño, J.M.; de Las Rivas, B.; Muñoz, R. Tannin degradation by a novel tannase enzyme present in some *Lactobacillus plantarum* strains. *Appl. Environ. Microbiol*. **2014**, *80*, 2991–2997. https://doi.org/10.1128/AEM.00324-14.
8. Pan, H.; Zhan, J.; Yang, H.; Wang, C.; Liu, H.; Zhou, H.; Zhou, H.; Lu, X.; Su, X.; Tian, Y. Improving the acid resistance of tannase TanBLp (AB379685) from *Lactobacillus plantarum* ATCC14917^T by Site-Specific Mutagenesis. *Indian J. Microbiol*. **2021**, *62*, 96–102 https://doi.org/10.1007/s12088-021-00983-x.
9. Esteban-Torres, M.; Landete, J.M.; Reverón, I.; Santamaría, L.; de las Rivas, B.; Muñoz, R. A *Lactobacillus plantarum* esterase active on a broad range of phenolic esters. *Appl. Environ. Microbiol*. **2015**, *81*, 3235–3242. https://doi.org/10.1128/AEM.00323-15.
10. Parente, E.; Grieco, S.; Crudele, M.A. Phenotypic diversity of lactic acid bacteria isolated from fermented sausages produced in Basilicata (Southern Italy). *J. Appl. Microbiol*. **2001**, *90*, 943–952. https://doi.org/10.1046/j.1365-2672.2001.01328.x.
11. Ciafardini, G.; Marsilio, V.; Lanza, B.; Pozzi, N. Hydrolysis of oleuropein by *Lactobacillus plantarum* strains associated with olive fermentation. *Appl. Environ. Microbiol*. **1994**, *60*, 4142–4147. https://doi.org/10.1128/aem.60.11.4142-4147.1994.
